# Association between vitamin D levels, hormonal profile, and ovarian reserve in infertile women

**DOI:** 10.1186/s12902-026-02500-x

**Published:** 2026-08-14

**Authors:** Maryam Azizi Kutenaee, Nasibeh Roozbeh, Elaheh Taghvaei, Ensieh Salehi, Roghayeh Ezati Rad

**Affiliations:** 1 Fertility and Infertility Research Center, Hormozgan University of Medical Sciences, Bandar Abbas, Iran; 2 Mother and Child Welfare Research Center, Hormozgan University of Medical Sciences, Bandar Abbas, Iran

**Keywords:** Vitamin D deficiency, Female infertility, Ovarian reserve, Luteinizing hormone, Metabolic-endocrine crosstalk

## Abstract

**Background and objective:**

The potential influence of vitamin D on female reproductive function has recently gained considerable attention, yet its connection with hormonal dynamics and ovarian reserve remains uncertain. This study explored how circulating vitamin D concentrations relate to reproductive hormones and ovarian reserve indices among infertile women.

**Methods:**

A retrospective cross-sectional study was conducted on medical records of 880 infertile women aged 18–45 years. Participants were grouped by serum vitamin D status as sufficient (≥ 30 ng/mL), insufficient (21–29 ng/mL), or deficient (≤ 20 ng/mL). Hormonal factors (FSH, LH, AMH, E2, TSH) and antral follicle count (AFC) were analyzed using Kruskal-Wallis and multinomial logistic regression.

**Results:**

Nearly half of the women (47.4%) were vitamin D deficient. Those with deficiency exhibited significantly greater BMI values and lower LH concentrations compared with women having sufficient vitamin D (*p* < 0.05). Regression modeling indicated that BMI positively predicted both insufficiency (OR = 1.045, *p* = 0.029) and deficiency (OR = 1.058, *p* = 0.001), whereas LH showed an inverse association with deficiency (OR = 0.964, *p* = 0.040). In adjusted analysis, higher AMH was associated with vitamin D insufficiency, whereas no significant association was observed for vitamin D deficiency.

**Conclusion:**

Vitamin D deficiency was common among infertile women and was associated with higher BMI and lower LH levels. These findings support a possible link between vitamin D status and selected metabolic and hormonal parameters; however, longitudinal studies are needed before clinical implications can be established.

## Background

 Infertility is a major public health problem, impacting approximately 15% of reproductive-age couples worldwide [1]. Beyond its clinical dimensions, infertility can lead to considerable emotional and social pain for individuals, couples, and communities [2]. According to global estimates, around 48 million couples—or nearly 186 million individuals—struggle with infertility [3]. In women, this condition may arise from a range of factors, such as hormonal imbalances, diminished ovarian reserve, lifestyle-related issues, and nutritional deficiencies [4]. Among these, vitamin D has emerged as a potentially relevant factor in female reproductive disorders in recent years [5].

Vitamin D is a seco-steroid hormone whose primary role is to maintain calcium and phosphate balance and bone metabolism [6]. Interestingly, researchers have discovered that vitamin D receptors (VDRs) are expressed in reproductive tissues including the ovaries, endometrium, and placenta—suggesting that its role extends well beyond skeletal health [7]. Adequate vitamin D status has been linked with reproductive mechanisms such as placental development, calcium transfer across the placenta, and implantation-related pathways, suggesting a broader role in female reproduction [8].

Beyond these functions, vitamin D also appears to play a regulatory role in ovarian activity. Vitamin D influences the expression of steroidogenic enzymes, enables the synthesis of estrogen and progesterone, and promotes follicular growth by acting on granulosa cells [9, 10]. Certain studies also suggest that vitamin D may influence the hypothalamic–pituitary–ovarian axis by affecting reproductive hormones such as AMH, FSH, and LH, which are relevant to ovarian function and reserve [11].

Some studies have reported a positive association between sufficient vitamin D status and higher AMH levels or improved ovarian reserve markers [11, 12], while others have not observed consistent associations with reproductive outcomes in infertility treatment settings [13]. To our knowledge, no comprehensive evaluation of the relationship between 25(OH)D and key reproductive hormones—including AMH, FSH, LH, E2, and TSH—has been conducted in the infertile population of southern Iran, a region where inhabitants share relatively uniform lifestyle conditions and comparable patterns of sun exposure due to similar climatic and cultural characteristics. This large-scale study, therefore, provides the first adjusted estimates of these associations in this understudied and relatively homogeneous population, offering adjusted associative evidence that may inform future hypothesis-driven and interventional research. In this context, the present research aims to examine the interaction between serum vitamin D levels, reproductive hormone patterns, and indices of ovarian reserve among infertile women. Better understanding of these associations may help generate hypotheses regarding potentially modifiable factors relevant to infertility care.

## Material & method

This study was designed as a retrospective cross-sectional study with the aim of assessing the relationship between serum levels of vitamin D, reproductive hormone profiles, and ovarian reserve indicators in infertile women. The study examined the data of infertile females who visited the center from January 2010 to January 2024 for infertility evaluation and treatment at Shahid Mohammadi Hospital, Bandar Abbas, Iran, which is a tertiary center. The patients were included according to available clinical records documented in the health information system of the clinic.

The inclusion criteria were: women aged 18–45 years, those presenting with primary or secondary infertility, having complete information on serum 25-hydroxyvitamin D [25(OH)D] and reproductive hormones (FSH, LH, Estradiol (E2), TSH, AMH). All serum measurements, including 25(OH)D and reproductive hormones (FSH, LH, E2, TSH, and AMH), were obtained on day 2 of the menstrual cycle during the initial infertility evaluation and recorded from the same clinical visit. Those subjects with ongoing or recent consumption (within 3 months) of vitamin D or hormonal supplements and incomplete or missing clinical details were excluded. All relevant clinical, demographic, and laboratory information was manually abstracted from patient charts and electronic medical records. The extracted variables included age, BMI, duration of infertility, serum 25(OH)D level, hormonal profile (FSH, LH, E2, TSH, AMH) and marker of ovarian reserve (antral follicle count (AFC)).

Statistical analysis was conducted with SPSS software (version 25.0, IBM Corp, Armonk, NY, United States). Descriptive statistics were employed to describe socio-demographic variables, hormonal profiles, and markers of ovarian reserve in three categories of vitamin D status: normal (≥ 30 ng/mL), insufficient (21–29 ng/mL), and deficient (≤ 20 ng/mL). Normality of continuous variables was assessed using the Shapiro-Wilk test, which revealed non-normal distribution for all hormone variables. Therefore, all continuous variables are presented as median with interquartile range (IQR). Group comparisons across vitamin D categories were performed using the Kruskal-Wallis test. Categorical variables were compared using the Chi-square test.

Multinomial logistic regression models were used to examine the relationship between vitamin D status and reproductive parameters. Two separate models were fitted: one between women with vitamin D insufficiency and normal, and the other between women with vitamin D deficiency and normal. Independent variables included age, BMI, FSH, LH, AMH, E2, TSH, and AFC. The findings were expressed as odds ratios (ORs) with their 95% confidence intervals (CIs), and *p* < 0.05 was taken as statistically significant. Prior to regression analysis, multicollinearity was assessed using variance inflation factors (VIF). Model adequacy was evaluated using likelihood ratio statistics, Pearson and Deviance goodness-of-fit tests, and pseudo-R² indices. A two-sided P value < 0.05 was considered statistically significant.

## Results

In this study, data on types of infertility were collected from 880 participants. The median (IQR) age of participants was 34 (29–40) years, with a median BMI of 26.5 (23–30) kg/m². The median (IQR) vitamin D level was 21.2 (15–31) ng/mL. Among the identified causes of infertility, female-related factors were responsible for 654 cases (74.3%), followed by male-related causes in 132 cases (15%), and combined male and female factors in 112 cases (12.7%). Within the female infertility subgroup, the most common etiology was unexplained infertility (21.6%), followed by diminished ovarian reserve (19%), and polycystic ovary syndrome (PCOS) (16.5%), respectively. Among the participants, 417 (48.3%) were vitamin D deficient, 207 (24%) had insufficient levels, and 256 (29.7%) had normal vitamin D levels. Primary infertility was the most common type, with 575 cases (65.3%) among the participants. Secondary infertility was observed in 152 cases (17.2%), and 153 women (17.4%) had a combination of both primary and secondary infertility. Table 1 presents the distribution of demographic, ovarian reserve, and hormonal parameters across vitamin D categories. Significant differences across vitamin D categories were observed for age, BMI, and LH (*p* < 0.05). No statistically significant between-group differences were found for AFC, AMH, FSH, E2, or TSH. To evaluate whether the relationship between vitamin D and AMH is modified by age or PCOS status, subgroup analyses were performed using the Kruskal-Wallis test. Stratification by age (< 35 vs. ≥35 years) revealed no significant difference in AMH levels across vitamin D categories in either age group (*p* > 0.05). Similarly, stratification by PCOS status (based on infertility etiology) showed no significant association between vitamin D and AMH in the PCOS group (*p* > 0.05).

In adjusted multinomial regression analysis in Table 2, higher BMI and higher AMH were independently associated with vitamin D insufficiency relative to normal vitamin D status. For each one-unit increase in BMI, the odds of being in the insufficient vitamin D group increased by 4.5% (OR = 1.045; 95% CI: 1.004–1.087; *p* = 0.029). Similarly, each one-unit increase in AMH level was associated with a 10% higher likelihood of vitamin D insufficiency (OR = 1.100; 95% CI: 1.010–1.199; *p* = 0.028). Other variables did not show a statistically significant association with vitamin D status.

The results of the multinomial regression analysis presented in Table 3 showed that vitamin D deficiency was significantly associated with BMI and LH levels. For each one-unit increase in BMI, the odds of vitamin D deficiency increased by 5.8% (95% CI: 1.022–1.095; *p* = 0.001). Conversely, there was a significant inverse association between LH levels and vitamin D deficiency, with each one-unit increase in LH associated with a 3.6% decrease in the odds of vitamin D deficiency (95% CI: 0.930–0.998; *p* = 0.040). Other variables did not show a statistically significant association with vitamin D status.

The multinomial logistic regression model demonstrated a significant improvement over the intercept-only model (Likelihood ratio χ² = 44.778, df = 16, *P* < 0.001). Goodness-of-fit statistics indicated an acceptable fit of the model to the observed data, with non-significant Pearson (χ² = 1450.279, df = 1424, *P* = 0.308) and Deviance (χ² = 1486.216, df = 1424, *P* = 0.123) tests. The pseudo-R² values were 0.060 (Cox and Snell), 0.068 (Nagelkerke), and 0.029 (McFadden). Multicollinearity diagnostics showed no evidence of problematic multicollinearity, with all variance inflation factor (VIF) values below 10.

**Table 1:** Socio-demographic, ovarian reserve and hormonal profile in normal, insufficiency and deficiency of vitamin D level in infertile women

Variable	Normal Vitamin D level (*N* = 256)	Insufficiency Vitamin D level (*N* = 207)	Deficiency Vitamin D level (*N* = 417)	*P*-value*
	Median (IQR)	Median (IQR)	Median (IQR)	
Age (years)	35 (29–40)	35 (30–40)	33 (28–38)	**0.022**
BMI (kg/m²)	25.45 (22.11–28.56)	26.50 (23.37–31.14)	27.05 (23.03–30.43)	**0.0004**
AFC (n)	13 (7–21)	12 (6–24)	14 (7–21)	0.207
AMH (ng/mL)	1.90 (0.98–3.82)	2.20 (1.06–5.10)	2.10 (1-4.60)	0.093
FSH (mIU/mL)	6.38 (5–9)	5.90 (4–9)	5.87 (4–8)	0.059
LH (mIU/mL)	4.60 (3–8)	4.09 (3–8)	3.80 (2.70-6)	**0.002**
E2 (pg/mL)	54 (33–75)	52 (34–93)	51.33 (36–74)	0.830
TSH (mIU/L)	1.96 (1.30–2.75)	2.04 (1.30–2.80)	2.04 (1.25–2.90)	0.769

*P-value based on Kruskal-Wallis test. Data presented as median (interquartile range, IQR). Bold values indicate statistical significance (*p* < 0.05)

**Table 2:** Multinomial regression of socio-demographic, ovarian reserve, and hormonal profile variables in women with vitamin D insufficiency compared with those with normal vitamin D levels

Variables	B	Std. Error	Sig.	OR	95% Confidence Interval	
					Lower Bound	Upper Bound
Intercept	-2.159	0.918	0.019	-	-	-
Age	0.016	0.019	0.395	1.016	0.979	1.055
BMI	0.044	0.020	**0.029**	1.045	1.004	1.087
AFC	− 0.009	0.017	0.595	0.991	0.959	1.024
AMH	0.096	0.044	**0.028**	1.100	1.010	1.199
FSH	− 0.004	0.015	0.803	0.996	0.968	1.025
LH	− 0.006	0.016	0.694	0.994	0.963	1.025
E2	0.000	0.001	0.664	1.000	0.999	1.002
TSH	0.066	0.072	0.355	1.069	0.929	1.230

Estimated unstandardized regression coefficients with 95% Confidence intervals are mentioned in this table. Bold values indicate statistical significance (*p* < 0.05). The normal vitamin D group served as the reference category

**Table 3:** Multinomial regression of socio-demographic, ovarian reserve, and hormonal profile variables in women with vitamin D deficiency compared with those with normal vitamin D levels

Variables	B	Std. Error	Sig.	OR	95% Confidence Interval	
					Lower Bound	Upper Bound
Intercept	0.219	0.789	0.782	-	-	-
Age	− 0.028	0.016	0.079	0.972	0.942	1.003
BMI	0.056	0.018	**0.001**	1.058	1.022	1.095
AFC	− 0.011	0.014	0.457	0.989	0.962	1.018
AMH	0.042	0.041	0.304	1.043	0.963	1.129
FSH	− 0.025	0.018	0.167	0.975	0.941	1.011
LH	− 0.037	0.018	**0.040**	0.964	0.930	0.998
E2	− 0.001	0.001	0.423	0.999	0.998	1.001
TSH	0.101	0.063	0.108	1.106	0.978	1.251

Estimated unstandardized regression coefficients with 95% Confidence intervals are mentioned in this table. Bold values indicate statistical significance (*p* < 0.05). The normal vitamin D group served as the reference category

## Discussion

In this cohort of infertile women, vitamin D deficiency and insufficiency were highly prevalent. The most consistent finding of the present study was an association between lower vitamin D status and higher BMI, which was observed both in crude comparisons and in adjusted multinomial regression models. In addition, vitamin D deficiency was inversely associated with LH levels. Although higher AMH was associated with vitamin D insufficiency in the adjusted model, this finding was not observed in crude comparisons, was not present in the deficiency model, and was not supported by subgroup analyses; therefore, it should be interpreted cautiously.

The high prevalence of low vitamin D levels observed in our study is consistent with previous reports in infertile women and women undergoing fertility evaluation [14, 15]. Given the expression of vitamin D receptors in reproductive tissues such as the ovary, endometrium, and placenta, vitamin D has been hypothesized to influence several reproductive processes, including follicular development, endometrial receptivity, and implantation [16, 17]. In support of this broader context, a Swedish cohort study also found a high frequency of low vitamin D levels among women undergoing IVF, with higher BMI and lower sun exposure identified as important correlates [18].

One of the most consistent findings in the present study was the association between lower vitamin D status and higher BMI. Women in the insufficiency and deficiency groups had higher median BMI values than those with normal vitamin D levels, and this association remained significant in the adjusted multinomial regression models. This finding is in line with previous studies showing an inverse relationship between adiposity and circulating vitamin D concentrations [19, 20]. A commonly proposed explanation is the sequestration of vitamin D in adipose tissue, which may reduce its bioavailability in the circulation. Because both obesity and vitamin D deficiency have been linked to impaired reproductive outcomes, their coexistence in infertile women may be clinically relevant [21]. However, due to the observational and cross-sectional nature of the present study, no conclusion can be drawn regarding the direction or mechanism of this relationship.

We also observed a significant inverse association between vitamin D deficiency and LH levels. Compared with women with normal vitamin D levels, those with deficiency had lower median LH concentrations, and this association remained significant after adjustment in the multinomial regression model. Although the biological basis of this relationship is not fully understood, prior evidence suggests that vitamin D may contribute to the regulation of gonadotropin secretion and ovarian steroidogenesis through effects on the hypothalamic-pituitary-gonadal axis [5]. Nevertheless, this finding should be interpreted with caution and confirmed in future prospective studies.

The relationship between vitamin D status and AMH was less clear. In the crude comparisons across vitamin D categories, AMH did not differ significantly, and no significant association was identified in subgroup analyses stratified by age or PCOS status. However, in the adjusted multinomial regression model, higher AMH was independently associated with vitamin D insufficiency relative to normal vitamin D status. This pattern suggests that the observed association may be sensitive to model specification and confounding structure rather than reflecting a robust and consistent biological relationship. The existing literature on vitamin D and AMH is similarly inconsistent. Some studies have suggested a positive association, possibly mediated through effects on granulosa-cell function and ovarian follicular activity [22], whereas others have found no significant relationship [23]. These discrepancies may be related to differences in study populations, infertility etiologies, ovarian reserve status, assay methods, vitamin D cut-off values, and unmeasured seasonal or lifestyle factors. Accordingly, the association between vitamin D status and AMH in the present study should be regarded as hypothesis-generating rather than conclusive.

In addition to endocrine pathways, vitamin D has also been implicated in inflammatory, oxidative, and immunomodulatory mechanisms that may influence female reproductive function [24, 25].

Although these pathways were not directly examined in the present study, they provide a possible broader biological context for understanding the multifaceted role of vitamin D in fertility. In the same vein, conditions such as PCOS may further modify the relationship between vitamin D and reproductive hormonal profiles. Previous studies have reported that vitamin D supplementation in women with PCOS may improve some metabolic and reproductive parameters [26–28]. Although PCOS was not the principal focus of the current analysis, its presence in a subset of participants may have contributed to heterogeneity in hormonal patterns.

This study has several limitations. First, serum vitamin D concentrations were measured at a single time point and may not fully reflect long-term vitamin D status. Second, although blood sampling for 25(OH)D and reproductive hormones was standardized and performed on day 2 of the menstrual cycle, potentially relevant factors such as sunlight exposure, dietary intake, sunscreen use, seasonal variation, and the exact season of blood collection were not available due to the retrospective design. Finally, the cross-sectional design precludes any causal inference; therefore, the observed associations should be interpreted as associative rather than causal.

## Conclusion

Vitamin D deficiency and insufficiency were common in this population of infertile women. Lower vitamin D status was consistently associated with higher BMI. In addition, vitamin D deficiency was inversely associated with LH levels, whereas higher AMH was associated only with vitamin D insufficiency in the adjusted model and was not consistent across analyses. Overall, these findings suggest that vitamin D status may be related to selected metabolic and hormonal parameters in infertile women; however, longitudinal and interventional studies are needed to clarify causality and clinical relevance.

## Data Availability

The datasets used and/or analyzed during the current study are available from the corresponding author on reasonable request.

## Abbreviations

25(OH)D: 25-hydroxyvitamin D
AFC: Antral follicle count
AMH: Anti-Mullerian hormone
BMI: Body mass index
CI: Confidence interval
E2: Estradiol
FSH: Follicle-stimulating hormone
IQR: Interquartile range
IVF: In vitro fertilization
LH: Luteinizing hormone
OR: Odds ratio
PCOS: Polycystic ovary syndrome
TSH: Thyroid-stimulating hormone
VDR: Vitamin D receptor
VIF: Variance inflation factor
